# Supplementary material for: Magnetization generation and giant nonlinear transport at symmetry-engineered interfaces
Source: Nat Commun. 2026 Jan 9;17:361. doi: 10.1038/s41467-025-66149-1 (PMC12796300; doi:10.1038/s41467-025-66149-1)
Supplement: Supplementary file 1 — Supplementary Information [file 41467_2025_66149_MOESM1_ESM.pdf]

Supplementary Information for

Magnetization generation and giant nonlinear transport at symmetry-engineered interfaces

Hang-Bo Zhang[1,7], Zhen-Yu Ding[2,7], Yi-Ning Xie[3], Zheng-Hao Li[1,4], Eoin Moynihan[3], Ana M. Sanchez[3], WenGuang Zhu[2,5,1], Yang Gao[2,5,1*], Yoshihiro Iwasa[6], Marin Alexe[3], Ming-Min Yang[1,4*]

[1]Hefei National Laboratory, Hefei, Anhui, 230088, China
[2]International Center for Quantum Design of Functional Materials (ICQD), Hefei National Research Center for Physical Sciences at the Microscale, University of Science and Technology of China, Hefei 230026, China
[3]Department of Physics, The University of Warwick, Coventry, CV4 7AL, UK
[4]School of Emerging Technology, The University of Science and Technology of China, Hefei, 230026, China
[5]Department of Physics, University of Science and Technology of China, Hefei, 230026, China
[6]Center for Emergent Matter Science (CEMS), RIKEN, Wako, Saitama, 351-0198, Japan
[7]These authors contributed equally: Hang-Bo Zhang, Zhen-Yu Ding
[*]Corresponding Author. Email: mingminyang@hfnl.cn; ygao87@ustc.edu.cn

Supplementary Note 1: (112)-$LaAlO_3/SrTiO_3$ sample characterization

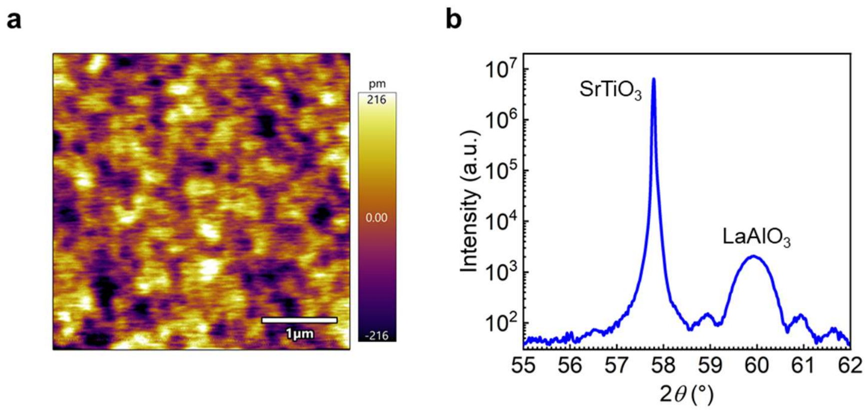

Fig. S1: Characterization of (112)-$LaAlO_3/SrTiO_3$ thin film quality. **a,** AFM mapping of sample topography. The RMS roughness is about 90 pm. **b**, X-ray diffraction of a (~10 nm) $LaAlO_3$ thin film grown on (112)-oriented $SrTiO_3$ substrate. Clear Laue oscillation peaks are revealed.

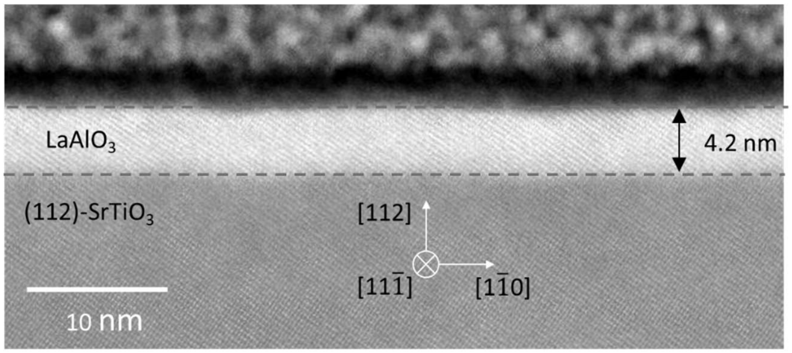

Fig. S2: Cross-sectional HAADF-STEM image of (112)-$LaAlO_3/SrTiO_3$ over a 54 nm region.

Supplementary Note 2: Discussion on the formation of the polar interface

It is worth noting that the "*polar conductive interface*" studied in our work can be defined as "a conductive interface with electrical carriers experiencing an electrical potential with an asymmetrical distribution, i.e. a potential gradient". Such an asymmetrical potential distribution is ubiquitous in the conductive interface in heterostructures. This is because, by the definition of "heterostructure", these two component layers have different chemical elements and thus, chemical potential. In the case of $LaAlO_3/SrTiO_3$ interface, the electrical carriers are confined in a narrow interface region of the $SrTiO_3$ side with no carriers in the insulating $LaAlO_3$ capping layer and the bulk of $SrTiO_3$ substrate. Therefore,

the distribution of electron density and the electrical potential are asymmetrical, i.e. polar. This has been theoretically discussed by W.-J. Son et al.[1], and P. Delugas et al.[2],, and then experimentally analyzed by J. Gabel et al[3].

In this sense, the "polar interface" discussed in our work is different but also more general than that related to the well-known "*polar catastrophe scenario*". In another word, any conductive interface with asymmetrical potential distribution can be treated as "polar interface", including but not limited to those induced by the "*polar catastrophe*". For example, the conduction layer induced in the field effect transistor can also be classified as the polar interface. In this regard, we fabricated a (112)-orientated metal-oxide field-effect transistor consisting of conductive $La_{0.3}Sr_{0.7}TiO_3$ layer and $SrTiO_3$ insulating layer, as illustrated in Fig. S3. The oxide layers of this structure are fabricated in 600 °C at a pressure of 0.01 mbar to exclude the introduction of oxygen vacancies in the $SrTiO_3$ substrate or the $SrTiO_3$ capping layer. The electron-dopped $La_{0.3}Sr_{0.7}TiO_3$ layer was about 10 nm and shows a metallic conduction. When a voltage of 0.4 V applied on the 10 nm-$SrTiO_3$ gating layer, electrons in the $La_{0.3}Sr_{0.7}TiO_3$ layer are attracted to the interface, enhancing the asymmetry of carrier distribution. As a results, a sizable nonlinear Hall effect has been then observed under this condition, with its magnitude 10 times larger than that without the applied voltage (Fig. S3). This case suffices the argument that a potential gradient is a general cause of the polarity, that are the origin of the physical effects discovered in this work.

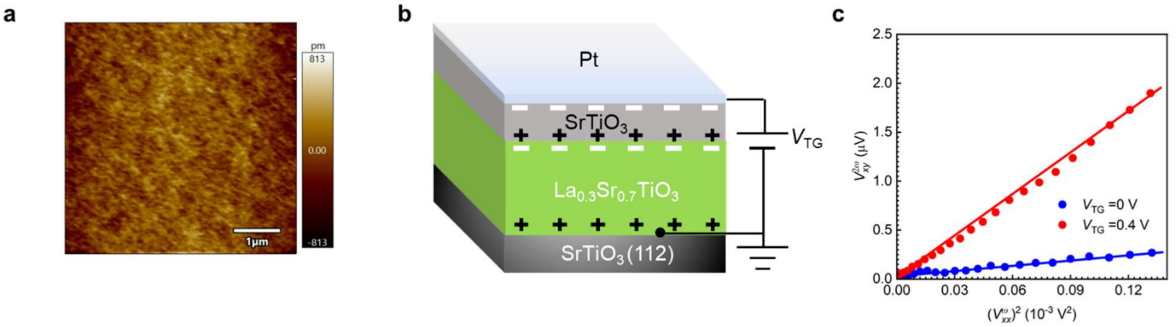

Fig. S3: Field effect to mimic a potential gradient-induced polarity. **a**, The illustration of (112)-orientated metal-oxide-semiconductor field effect transistors, $La_{0.3}Sr_{0.7}TiO_3$ layer is about 10 nm. **b**, AFM topology of (112)-orientated $La_{0.3}Sr_{0.7}TiO_3/SrTiO_3$.**c**, The corresponding NLHE effects measured in the direction $[1\bar{1}0]$ with and without a bias.

We also show here the layer-resolved effective charges distribution across $LaAlO_3/SrTiO_3$ interface. It demonstrates the similar profile as the (110)-$LaAlO_3/SrTiO_3$ system reported[4,5]. Based on this simple

ionic schematic, the ‘polar discontinuity’ does not exist. However, interfaces are conductive with a metallic behaviour till low temperature as the same situation in this work. Thus, our result suggests that a simple catastrophe model cannot explain the formation of the 2DEG at the interface.

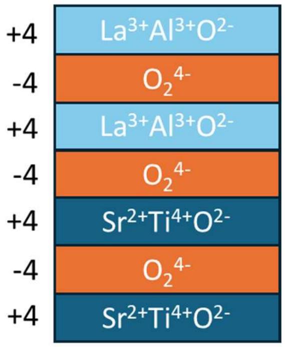

Fig. S4: Layout of ‘polar catastrophe’ model for (112) interfaces between $LaAlO_3$ and $SrTiO_3$.

Supplementary Note 3: Discussion on the mirror plane $M_{[1\bar{1}0]}$ in (112)-$LaAlO_3/SrTiO_3$ system

In an ideal situation, the designed mirror plane $M_{[1\bar{1}0]}$ at the (112)-interface can persist only if the (112)-interface is perfect without any defects. Any imperfections of the interface that are inevitable in the experimental process, such as miscut induced by the polishing process, interface interdiffusion, interlayer strain, etc., would impact the $M_{[1\bar{1}0]}$ mirror plane to a certain extent. However, these imperfections happen in a random and uncontrollable manner. Thus, their perturbation on the mirror plane and the associated physical effects would be reduced to a negligible level once summed over a large area, such as a Hall bar channel. This is confirmed by three evidences in our work. (1) If these imperfections would break the in-plane mirror symmetry and significantly modulate interface properties, we would have observed the nonlinear Hall effect at (001), (110), (111)-oriented interface. Clearly, this is not the case as shown in our work (Fig. 3d). (2) If the $M_{[1\bar{1}0]}$ is significantly broken as happened in $M_{[110]}$ and $M_{[001]}$, there would also be substantial nonlinear Hall effect and current-induced magnetization when the current is flowing along the $[11\bar{1}]$ in-plane direction. This is again not the case in our work. (3) We have also characterized the interface structure over a 54 nm width by STEM (Fig. S2), which shows the interface is flat within a 0.5 nm fluctuation without defects occurring

in an ordered manner. Therefore, based on the group theory argument and the behaviours of the physical effect at the (112)-interface, it is reasonable to claim the preservation of the only mirror plane $M_{[1\bar{1}0]}$ and thus its symmetry as C_s.

Supplementary Note 4: Ordinary Hall effect and carrier mobility anisotropy

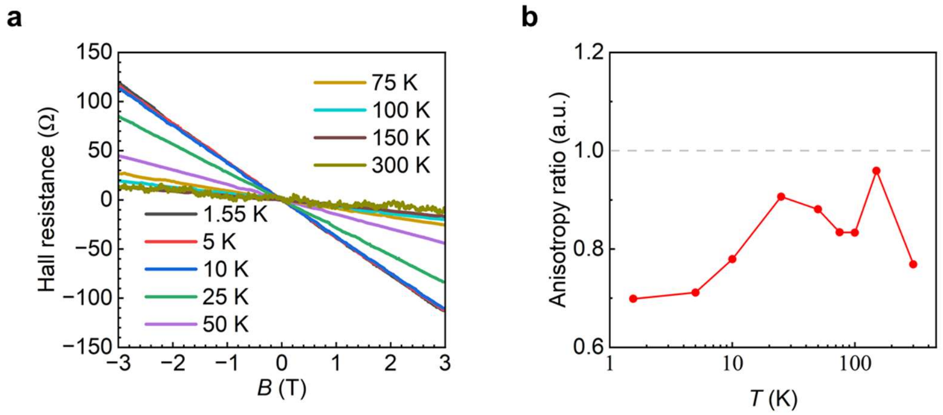

Fig. S5: Hall measurement and carrier mobility. **a,** Ordinary Hall effects measured in the direction $[1\bar{1}0]$ with the magnetic field up to ± 3 T. **b,** The carrier mobility anisotropy $u_s^{[11\bar{1}]}/u_s^{[1\bar{1}0]}$ is inconsistent with the resistance anisotropy.

Supplementary Note 5: Circular photogalvanic effect study

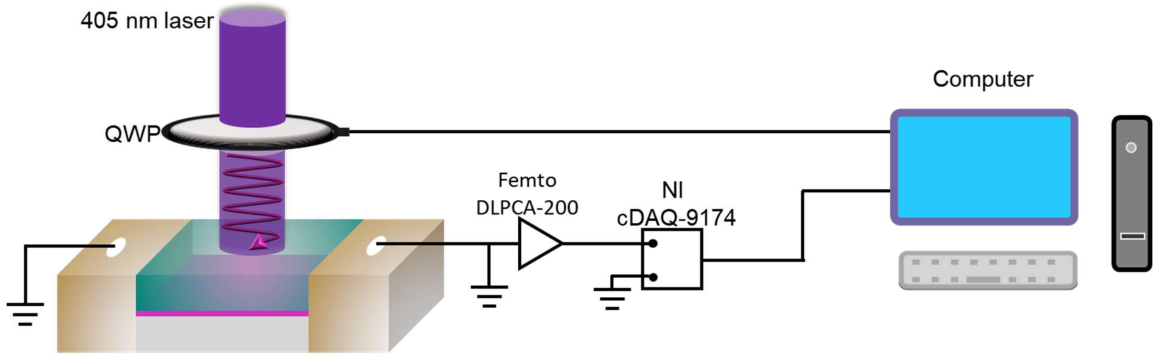

Fig. S6: The sketch of the CPGE measurement setup. A 405 nm (3.06 eV) laser with an intensity of ~37 mw was used to illuminate at normal incidence the sample surface. The linearly polarised light is transformed into circularly polarised light by QWP. The photocurrent passing the amplifier was converted to the voltage signal and further collected by the data

acquisition instruments. The computer controls the rotation of QWP and simultaneously takes the data of DAQ.

We define the angle of the quarter-wave plate, θ^{QWP}, as the angle between the fast axis of the QWP and the linear polarization direction of the incident beam, where the polarization direction is fixed along the laboratory axis x^{Lab}, as seen in Fig. S7. The sample is placed with its [112] direction parallel to the light propagating direction, i.e., the z^{Lab}. We address here that, the sample's in-plane crystallographic direction is usually not aligned with x^{Lab} or y^{Lab}, there is an angle offset θ^{Off}. This angle offset θ^{Off} appears in the measured and fitted curve of photocurrent vs QWP angle.

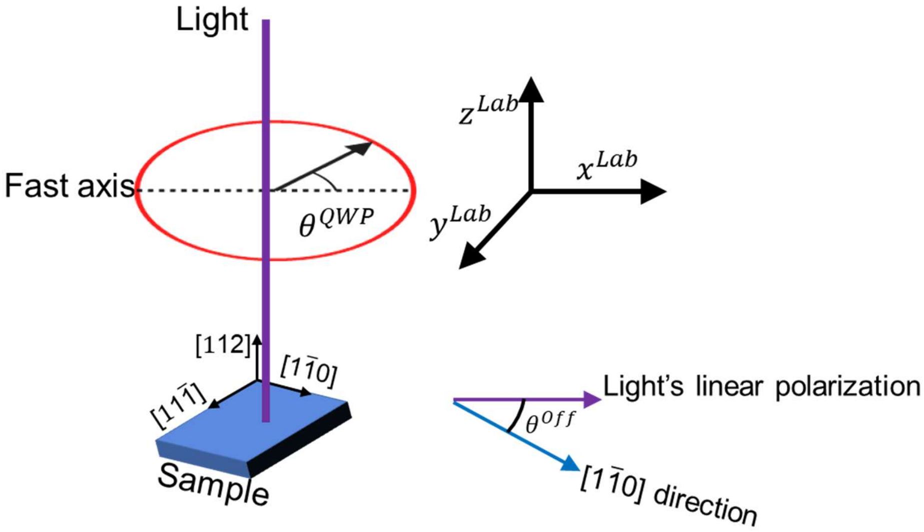

Fig. S7 Relationship between the QWP angle and the crystallographic direction.

The fitting results of the phocurrent as a function of QWP angle are in good agreement with the experimental data, as shown in Fig. S8 and Table S1.

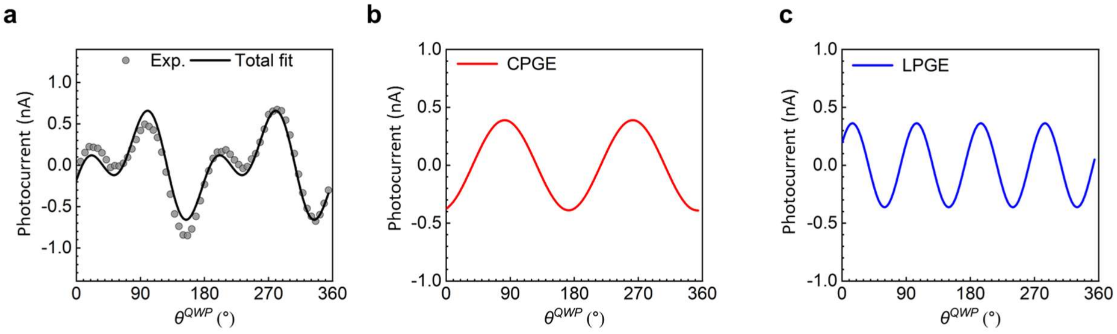

Fig. S8 Fitting results of the photocurrent in direction [1$\bar{1}$0]. a, Experimental data and the total fit of photocurrent vs θ^{QWP}. **b**, CPGE component. **c**, LPGE component.

Table S1 Fitting parameters of CPGE in direction [$1\bar{1}0$]

Fitting parameters	Abs. value	Standard error
CPGE component	0.372 nA	0.0197 nA
LPGE component	0.392 nA	0.0197 nA
θ^{Off}	52.88°	0.669°
R^2	0.93	N/A

Supplementary Note 6: Magneto-optic Kerr effect measurement

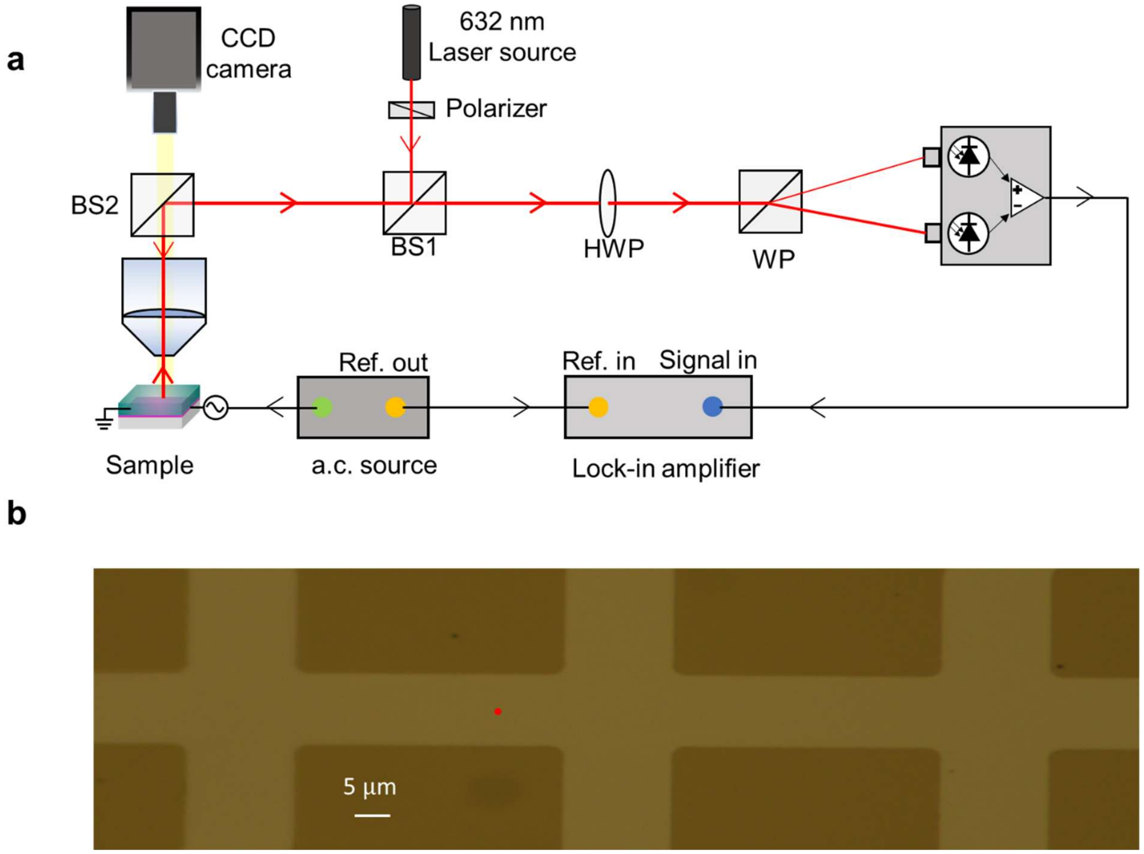

Fig. S9: Polar MOKE measurement. **a**, Schematic illustration of the polar-MOKE microscope set-up. The red laser beam

(632 nm) first passes through a polarizer and then a beamsplitter (BS1) (its direction is indicated by the thinner red triangle arrows), then is reflected by a beam splitter (BS2) and arrives at the sample through an objective. After being reflected by the sample, the light is further reflected by the same BS2 and then passes the same BS1 (its direction is indicated by a thicker arrow). Then, the reflected beam goes through a half wave plate (HWP) and a Wollaston prism (WP), which are placed before the auto balanced detector. An a.c. current from a current source is input to the conductive Hall bar channel, and its synchronous reference signal is fed into a lock-in amplifier for accurate signal extraction. **b**, Optical microscopy image of the Hall bar channel for MOKE measurement. The laser spot is about 1 μm, indicated by the red dot.

Supplementary Note 7: MOKE study in a (110)-$LaAlO_3/SrTiO_3$ thin film

Interfaces with mirror planes more than 1 do not show current-induced out-of-plane magnetization due to the symmetry constraint. To verify this, we conducted the MOKE study a (110)-$LaAlO_3/SrTiO_3$ thin film, which has the vanishing MOKE signal.

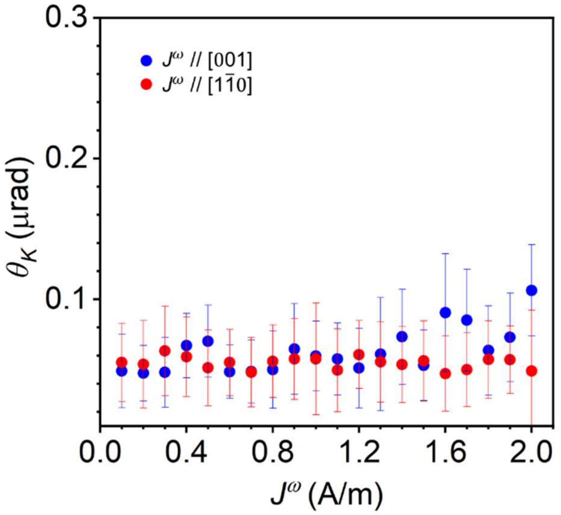

Fig. S10: MOKE study in a (110)-$LaAlO_3/SrTiO_3$ sample. Kerr rotation θ_K as a function of current amplitude measured in directions of [001] and [1$\bar{1}$0] of sample (110)-$LaAlO_3/SrTiO_3$.

Supplementary Note 8: Details of electrical measurement on Hall bar and disc devices

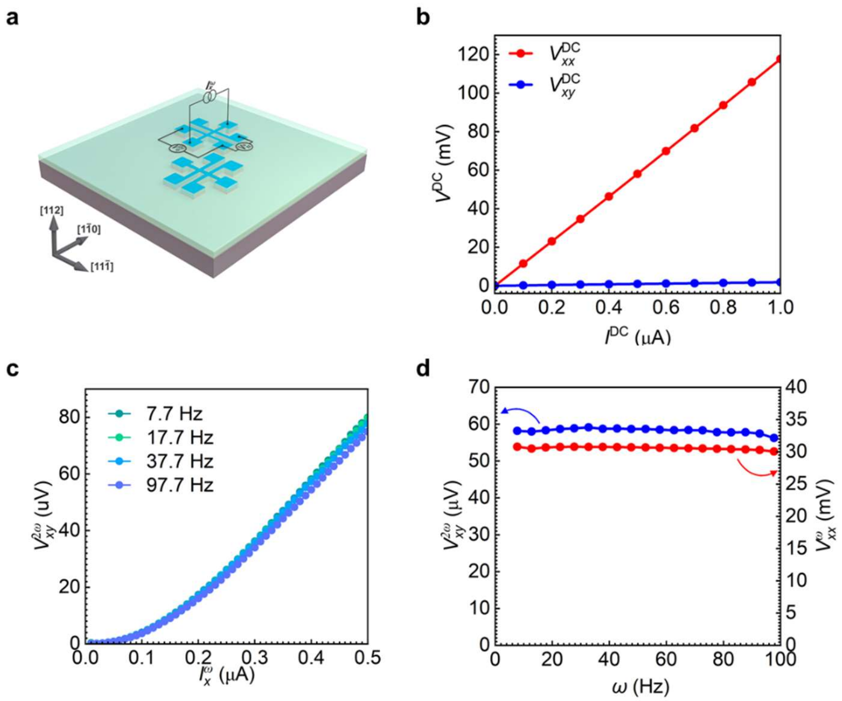

Fig. S11: Electrical measurement using a Hall bar device. a, Configuration of measurement in a Hall bar device **b**, DC voltages V_{xx} and V_{xy} as a function of d.c. current I^{DC} along $[1\bar{1}0]$ direction. The value of V_{xy} is only about 1% of V_{xx}, indicating the good alignment of the Hall bar device. **c**, $V_{xy}^{2\omega}$ as a function of driving current amplitude at various frequencies 7.7 Hz, 17.7 Hz, 37.7 Hz and 97.7 Hz measured in direction $[1\bar{1}0]$. **d**, Second harmonic transverse voltage $V_{xy}^{2\omega}$ and first harmonic longitudinal voltage V_{xx}^{ω} both measured in direction $[1\bar{1}0]$ as a function of driving frequency. The driving current amplitude is kept constant, that is 0.4 μA.

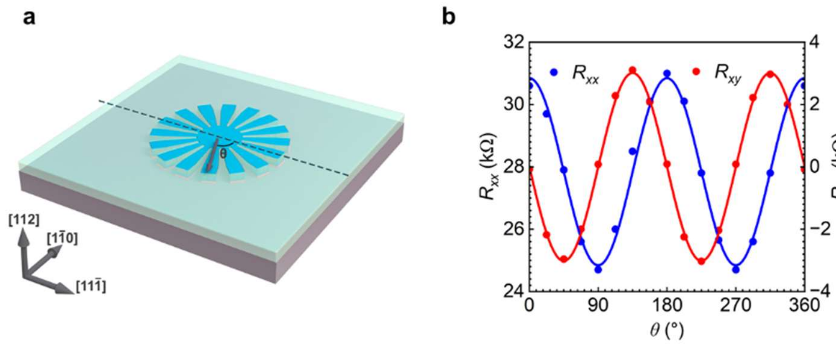

Fig. S12: Multi-directional electrical measurements using a disc device. a, Illustration of the circular disc device used to study the NLHE as a function of angle θ, defined as the angle between the bias current and the $[11\bar{1}]$ crystallographic direction. **b**, Resistances R_{xx} and R_{xy} of as a function of the angle θ. Dots and solid lines are experimental data and their fits (see Methods), respectively.

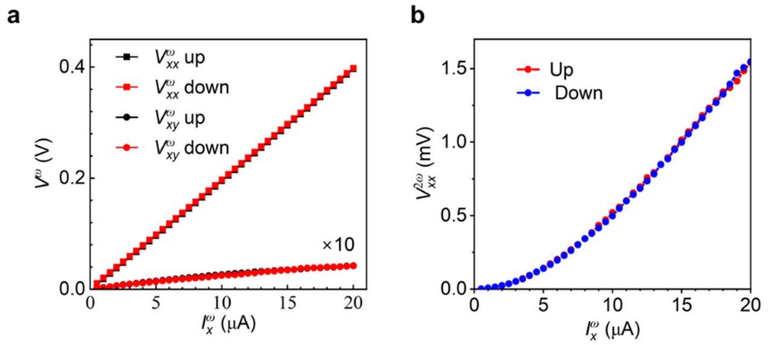

Fig. S13: Electrical measurement of a Hall bar device in a large ac current range. **a**, The 1st order voltage drops V_{xx}^{ω} and V_{xy}^{ω} and **b**, the 2nd order voltage drop $V_{xy}^{2\omega}$ as a function of ac current up to 20 μA.

Supplementary Note 9: Comparison of nonlinear Hall effects between different materials

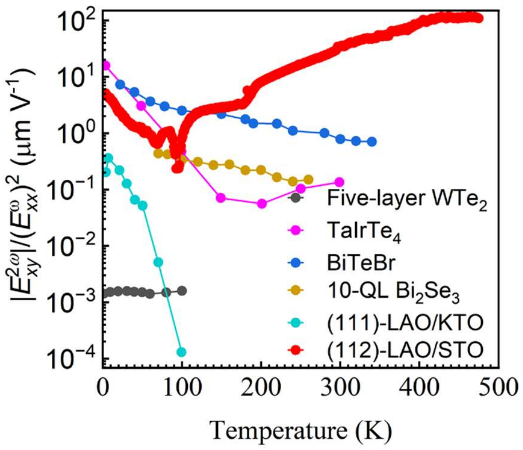

Fig. S14: Comparison of nonlinear Hall coefficients of different materials. The presented materials are five-layer WTe_2[6], $TaIrTe_4$[7], BiTeBr[8], 10-quintuple layer (QL) Bi_2Se_3[9], (111)-orientated $LaAlO_3/KTaO_3$ ((111)-LAO/KTO)[10] and our work (112)-$LaAlO_3/SrTiO_3$.

Supplementary Note 10: Discussion on the scaling law of NLHE

It has been reported that, in addition to the intrinsic contribution from the BCD, extrinsic contributions, including side jumping and screw scattering, play an important and even dominant role in the NLHE.

To resolve various contributions, we employ the scaling method at both low (< 30 K) and high temperature (> 100 K) ranges (Fig. S15). The two curves in Fig. S15 are obtained by relating NLHE coefficient $E_{xy}^{2\omega}/(E_{xx}^{\omega})^2$ and conductivity σ_s at each temperature. According to the theory[11], the scaling law for NLHE can be written as: $E_{xy}^{2\omega}/(E_{xx}^{\omega})^2 = A_1\,(\frac{\sigma_s}{\sigma_0})^2 + A_2\frac{\sigma_s}{\sigma_0} + A_3$, where A_1, A_2, A_3 are the scaling parameters, σ_0 is the conductivity at 1.55 K. The fitting results of A_1, A_2, A_3 are shown in Table S1. Based on the NLHE scaling law, the scaling parameters are expressed as:

$A_1 = C^{sk,2} + (C_{00}^{sk,1} + C_{11}^{sk,1} - C_{01}^{sk,1})$	(S.1)
$A_2 = C_{01}^{sk,1} - 2C_{11}^{sk,1} + C_0^{sj} - C_1^{sj}$	(S.2)
$A_3 = C_{in} + C_1^{sj} + C_{11}^{sk,1}$	(S.3)

where C_{in} is intrinsic contribution, C_i^{sj} the side-jump, $C_{ij}^{sk,1}$ the Gaussian skew-scattering, $C^{sk,2}$ the non-Gaussian skew-scattering, and i, j = 0 (1) denotes the defects (phonon) scattering source. There are seven parameters, i.e., seven different contributions in above three equations, which is impossible resolve quantitatively. To get a tentative insight into these contributions, here we take a bold but almost reasonable assumption.

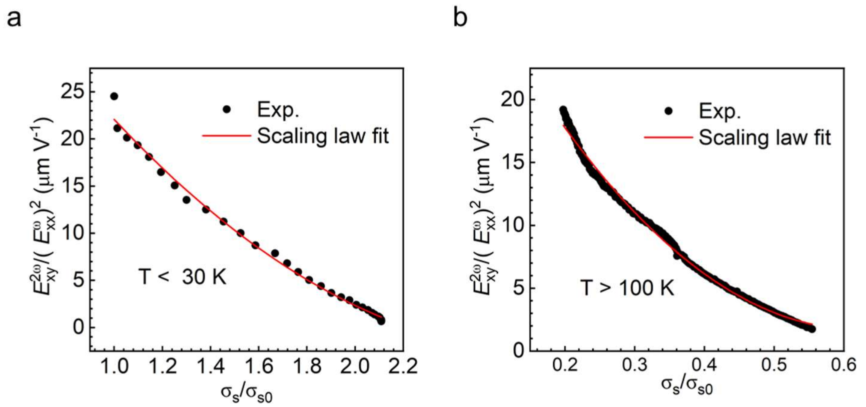

Fig. S15: Scaling law analysis of the NLHE at temperature. (a) lower than 30 K and (b) higher than 100 K.

Table S2: Scaling parameters

	A_1	A_2	A_3
T < 30 K	7.56	-42.4	56.9
T > 100 K	90.5	-112.4	36.6

Since $C^{sk,2}$ only exist in the A_1 parameter, the non-Gaussian skew-scattering only plays a secondary role. In the temperature range below 30 K, the parameter A_1 is almost one order of magnitude smaller than A_2 and A_3. This indicates that the screw scattering contributions play a minor role at low temperatures. Also, the phonon density is largely suppressed at low temperatures; it would be reasonable to assume C_0^{sj} (i.e., side jump due to defects) is much larger the C_1^{sj} (i.e., side jump due to phonons). Thus, $A_2 = -42.4 \approx C_0^{sj}$. Similarly, $A_3 = 56.9 \approx C_{in}$.

In the temperature range above 100 K, the parameter A_1 shares a similar magnitude with A_2 but with opposite sign, while A_3 is about three times smaller. Based on equations (S1) and (S2), the opposite sign between A_1 and A_2 is most likely due to the opposite sign of $C_{11}^{sk,1}$ and $C_{01}^{sk,1}$ in these two equations. Since $C_{00}^{sk,1}$(screw scattering due to defects) only exist in A_1, it may play a minor role here. Moreover, since side jump process is insensitive to the temperature, we assume C_0^{sj} remains as a constant over the whole temperature range. Thus, equations (S1-S3) can be approximated as:

$A_1 = C_{11}^{sk,1} - C_{01}^{sk,1} = 90.5$	(S4)
$A_2 = C_{01}^{sk,1} - 2C_{11}^{sk,1} - C_1^{sj}$ =-70	(S5)
$A_3 = C_{in} + C_1^{sj} + C_{11}^{sk,1} = 36.6$	(S6)

Thus, we get $C_{in} = 57$, which is almost equal to that derived at low temperature range, supporting above approximation.

Supplementary Note 11: Discussion on the temperature dependence of NLHE

Based on the above analysis, we can reach here a qualitative understanding of the origins and temperature dependence of the NLHE in the (112)-$LaAlO_3$/$SrTiO_3$ interface. Clearly, both the intrinsic and extrinsic contributions play important and compensating roles in this system. For example, the extrinsic contribution $C_1^{sj} + C_{11}^{sk,1}$ $(= -20.4)$ is opposite to that induced by Berry curvature dipole ($C_{in} = 57$). The peculiar temperature dependence can be ascribed to the role of the parameter A_2, i.e., the contribution of screw scattering and side jump. This also means that the NLHE effect of the (112)-

interface is highly related to its conductivity with an approximated quartic dependence. When the temperature decreases from 300 K to 100 K, the conductivity and mobility increase, the screw scattering process is enhanced, inducing a negative contribution that compensates the contribution of the intrinsic one. At temperatures below 50 K, the density of phonons is significantly reduced and thus, the related screw scattering contributions ($C_{01}^{sk,1}$ and $C_{11}^{sk,1}$) are suppressed. In this circumstance, the side jump and intrinsic effect dominate. With further decreasing temperature towards 1.55 K, the conductivity decreases, the compensating contribution of side jump by defects are reduced, leading to an enhanced NLHE.

Supplementary Note 12: Nonlinear Hall effects, AFM and XRD characterization of $LaAlO_3/SrTiO_3$ with different orientations

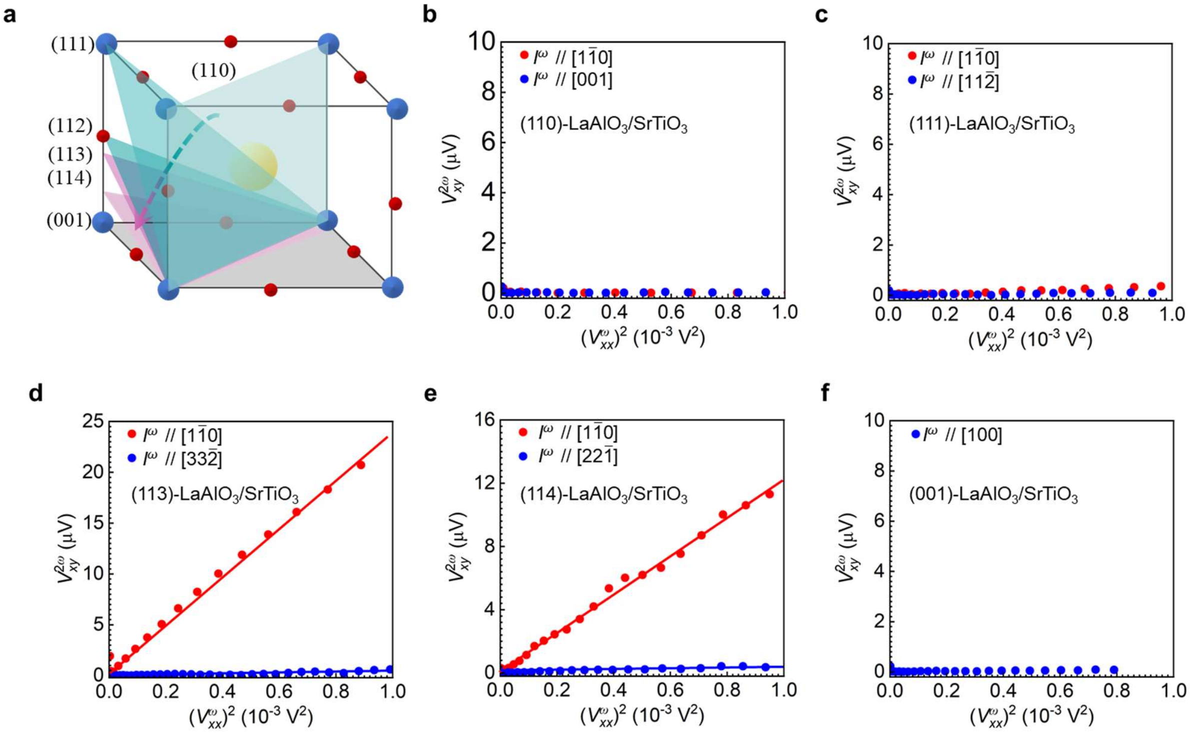

Fig. S16: Effect of substrate orientation on the NLHE of the $LaAlO_3/SrTiO_3$ system. **a**, Illustration of $SrTiO_3$ substrates with different high index crystallographic cuts employed to study the influence of symmetry. **b-f** $V_{xy}^{2\omega}$ voltage as a function of the V_{xx}^{ω} of (**b**), (110)-$LaAlO_3/SrTiO_3$, (**c**), (111)-$LaAlO_3/SrTiO_3$, (**d**), (113)-$LaAlO_3/SrTiO_3$, (**e**), (114)-$LaAlO_3/SrTiO_3$ and (**f**), (001)-$LaAlO_3/SrTiO_3$. The NLHE of (112)-$LaAlO_3/SrTiO_3$ thin film is given in the main text. The corresponding currents biased in these measurements are 1 μA.

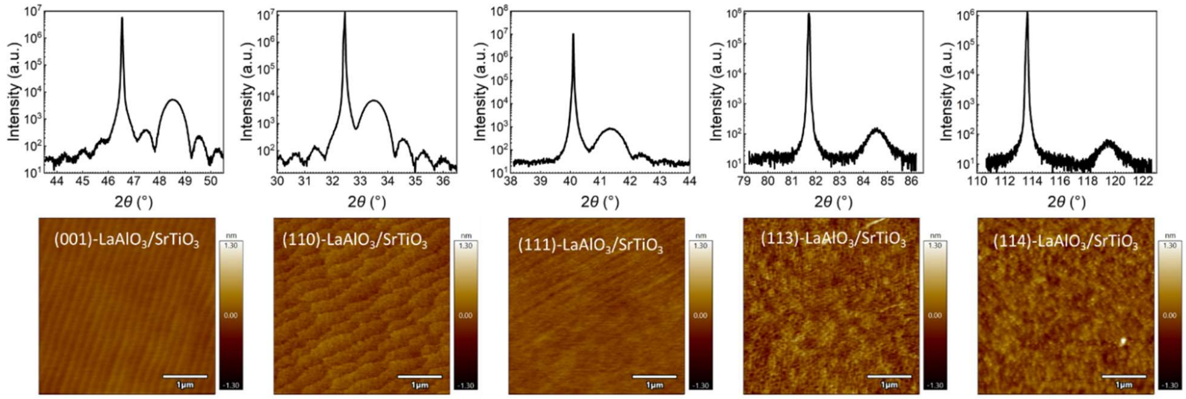

Fig. S17: XRD (upper panel) and AFM (lower panel) characterizations of (001)-, (110)-, (111)-, (113)-, (114)-$LaAlO_3/SrTiO_3$ thin films.

Supplementary Note 13: Role of zigzag patterns and defects

We first analyze the potential zigzag patterns at the interface as shown in Fig. S18. In the (112)-oriented $SrTiO_3$ interface/surface possesses only one in-plane mirror plane (i.e. $M_{[1\bar{1}0]}$). This might explain why the zigzag pattern is more obvious in the STEM image of (112)-orientated substrate than that of the (110) one. Such a low symmetry enforces the interface/surface to show zig-zag pattern as illustrated in Fig. RR2.5b wherein the step edge also shows a zig-zag pattern.

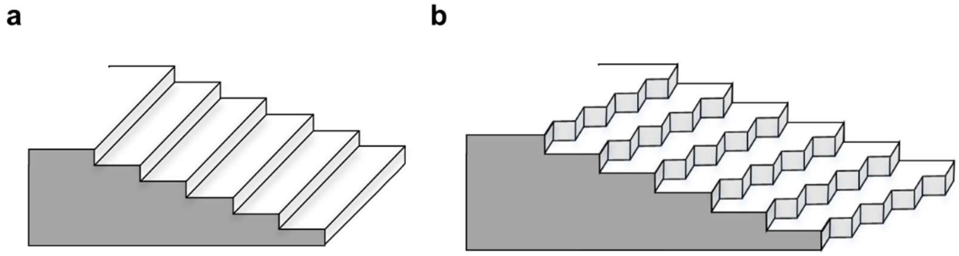

Fig. S18 Zigzag pattern configurations. (a) (110)-orientated surface. (b) (112)-orientated surface.

Although we have not observed regular zig-zag pattern in our (112)-$LaAlO_3/SrTiO_3$ sample over a large scale in the surface topography characterized by AFM, we believe such a zig-zag pattern (if manifests) also plays a role to the observed effect. For this, we specifically fabricated two $SrRuO_3/SrTiO_3$ thin films with different growth condition. *Sample #1*: (112)-$SrRuO_3/SrTiO_3$ with defects was formed in $SrRuO_3$. Nominal 8 u.c. $SrRuO_3$ is grown on (112)-$SrTiO_3$ at 800 ℃ at an oxygen pressure of 10^{-4} mbar, mimicking the growth condition of 2DEG (112)-LAO/STO. *Sample*

#2: (112)-$SrRuO_3/SrTiO_3$ with minimised defects formed in $SrTiO_3$. Nominal 8 u.c. $SrRuO_3$ is grown on (112)-$SrTiO_3$ at 600 °C at an oxygen pressure of 0.13 mbar to largely reduce the oxygen vacancies. It shows in Fig. S19 that either sample could present a sizable NLHE or MOKE signal. This indicates that the potential octahedral rotation is not a main factor influencing the physical properties observed.

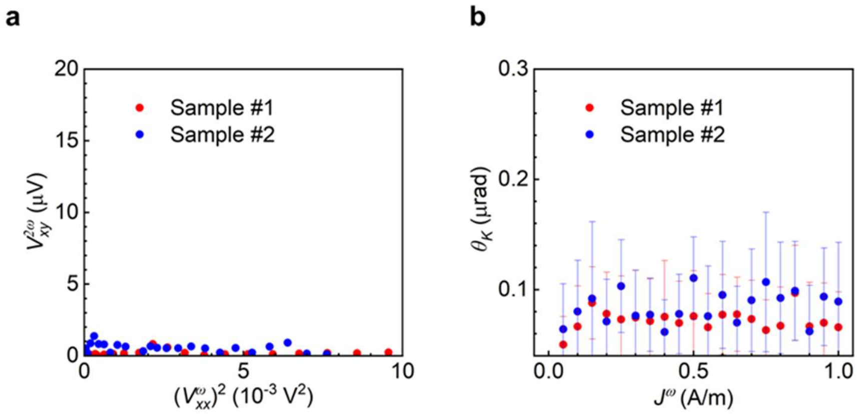

Fig. S19 NLHE and MOKE study of two (112)-$SrRuO_3/SrTiO_3$ samples. (a) NLHE comparison. (b) MOKE signal comparison.

Supplementary Note 14: DFT modeling and the relationship between carrier density and Fermi level

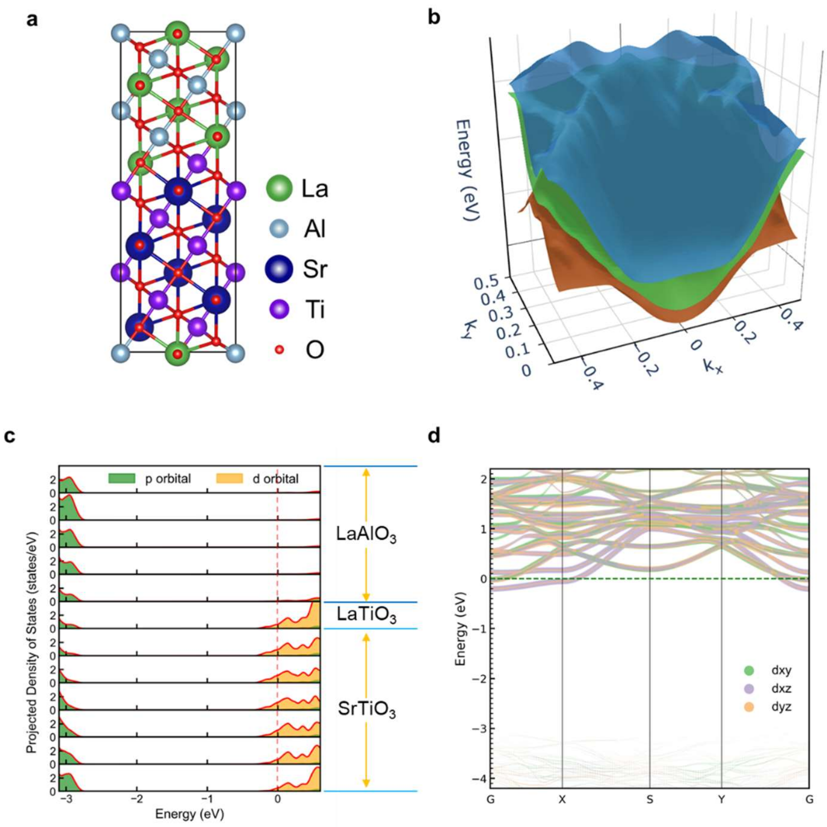

Fig. S20: Orbital-projected DFT calculations of the (112)-LaAlO3/SrTiO3 superlattice. a, The superlattice model used in DFT calculations. **b**, 3D band structure near the Fermi level. **c**, The layer-resolved projected density of states (PDOS). The results indicate that the electronic states near the Fermi level in the interface region predominantly originate from the d-orbitals of Ti atoms on the $SrTiO_3$ side. **d**, Fat-band diagrams of Ti d_{xy}, d_{xz} and d_{yz} orbitals. The results explicitly demonstrate in k-space that the conductive states are primarily derived from the Ti d-orbitals.

The Fermi level position is highly related to the carrier density. The carrier density of our sample, as presented in Fig. 2a in the main text, decreases with the temperature and saturates out in the low temperature limit, with its magnitude in the order of $10^{13} - 10^{14}$ cm^{-2} in the whole temperature range. These features are similar to other reported $LaA3O_3/SrTiO_3$ systems[5,12]. In order to unveil the relationship between the carrier density and Fermi level shift, we conducted the DFT calculations, as shown in Fig. S21. At the pristine Fermi energy, the carrier density is approximately 2.6×10^{14} cm^{-2}. As the Fermi level is raised by +0.1 eV, the carrier density increases to 5.3×10^{14} cm^{-2}; conversely, lowering the Fermi level by -0.1 eV results in a reduced density of 1.1×10^{14} cm^{-2}. Although other effects might also affect the carrier density, the present calculations imply a strong connection between the Fermi level and carrier density. We also would like to address here that the calculated carrier density is based

on the ideal model at zero temperature and might diverge from the real situation, as the experimentally observed carrier density at low temperature (such as ~2 K) is usually at the order of 10^{13} cm^{-2}.

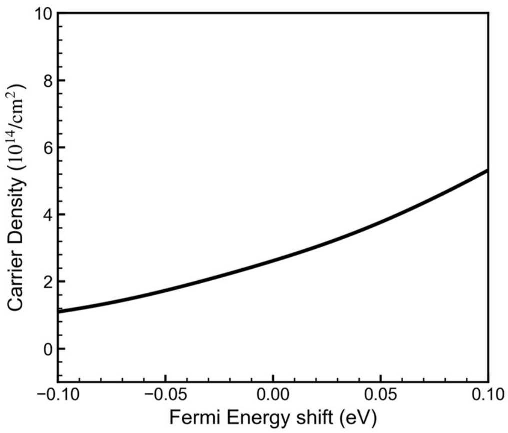

Fig. S21: Calculated 2D carrier density versus Fermi level shift in the (112)-$LaAlO_3/SrTiO_3$ heterostructure.

Supplementary Note 15: Evaluation of spin-orbit coupling effect on Berry curvature and Berry curvature dipole

The spin–orbit coupling (SOC) effect is important to the anomalous Hall effect as reported in the literature[13,14]. However, in the nonlinear Hall effect induced by BCD, the SOC effect is not the main contribution, such as in the work of twisted Bilayer Graphene[15,16] and WTe_2[17]. In our $LaAlO_3/SrTiO_3$ interface, we note here that the SOC effect and the associated spin contribution do not play a critical role. In 2023, M. T. Mercaldo et al. reported that orbital degrees of freedom combined with crystal field effects can intrinsically generate strong Berry curvature (BC) features in the 2D electron gas system, such as the $LaAlO_3/SrTiO_3$ interface, in the absence of spin–orbit coupling[18]. Later, E. Lesne et al. experimentally studied the nonlinear transport and BC features of (111)-oriented $LaAlO_3/SrTiO_3$ interface at low temperature[12]. Their results show orbital-sourced Berry curvature dipole is two orders of magnitude larger than the spin-sourced one, indicating a negligible role of the SOC effect in the nonlinear Hall effect. Regarding the charge-to-magnetization conversion, Johansson et al. showed theoretically that the orbital Rashba conversion efficiency should dominate in this system and be at least an order of magnitude larger than the spin effect[19]. Experimentally, A. E. Hamdi et al. showed that the orbital contribution dominates in the charge-magnetization conversion process of the (001)-$LaAlO_3/SrTiO_3$ interface, which is about 16 times the spin effect[20]. Overall, previous research on the

LaAlO3/SrTiO3 interface supports the dominant contribution of orbital degree of freedom and a minor role of the SOC effect.

To evaluate the role of SOC in our system, we first analyzed the band structures of the (112)-$LaAlO_3/SrTiO_3$ heterostructure, focusing on the electronic states near the Fermi level that are very related to transport properties and BC effects. For the Berry curvature dipole (BCD) under investigation in this work, its behaviour is critically dependent on the electronic structure information within a very narrow energy range near the Fermi level, which is clearly demonstrated in the formula

$$D_{\alpha\beta} = \int [d\mathbf{k}] D_{\alpha\beta}(\mathbf{k}) = \int [d\mathbf{k}] \sum_n \frac{\partial E_n}{\partial k_\alpha} \Omega_n^\beta \delta(E_n - \mu) \,. \qquad \text{(S7)}$$

As shown in Fig. S22a, we have shown the energy bands within the range of ± 0.2eV around the Fermi level along the $\Gamma - X$ path. The band splitting depicted here is caused by SOC. It can be observed that the band splitting is less than 10 meV across the entire plotting range, contributing the maximum band splitting (~10 meV) at an energy 0.13 eV above the Fermi level. In Fig. S22b, we have magnified the region where the bands cross the Fermi level, revealing a band splitting of approximately 2 meV. These results indicate that the contribution of SOC to the nonlinear Hall transport behaviour in (112)-$LaAlO_3/SrTiO_3$ is weak, especially at room temperature.

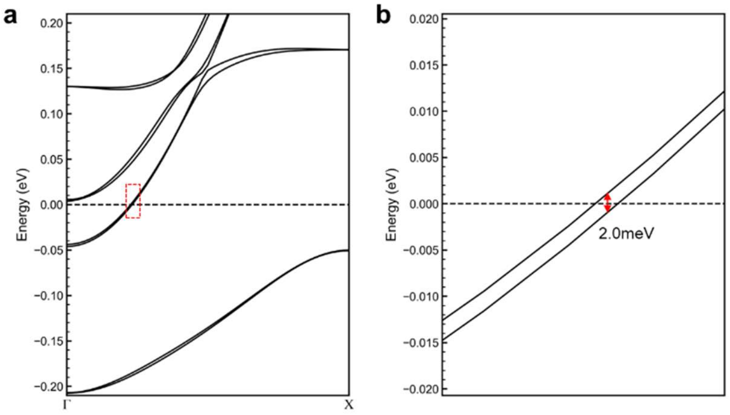

Fig. S22: SOC-induced band splitting near the Fermi level in the (112)-$LaAlO_3/SrTiO_3$ heterostructure. a, band structure along the Γ–X direction near the Fermi level. The red dashed box highlights the region of interest. **b,** Magnified view of the boxed region in (a), showing a spin-orbit-induced band splitting of approximately 2.0 meV near the Fermi energy, as indicated by the red arrow.

To elucidate the contribution of SOC effect on the geometrical feature of the (112)-interface, we have

calculated the BC and BCD features without the SOC effect and compared them to that with the SOC effect (as shown in Fig. S23). Without the SOC effect, the BC and BCD shows spin-degenerate features. The integrated BCD without the SOC effect shows a similar magnitude to that taking SOC effect into consideration. On the other hand, the SOC effect lifts the spin degeneracy and induces anti-crossing features in the band structure, which modifies the dependence of the BCD on the Fermi level position. Thus, our calculation indicates the major contribution of the BCD in the (112)-$LaAlO_3$/$SrTiO_3$ interface arises from the orbital degree of freedom, whereas the SOC effect, which activates the spin-contribution, modifies the quantum geometrical feature in a minor way. This is consistent with previous works.

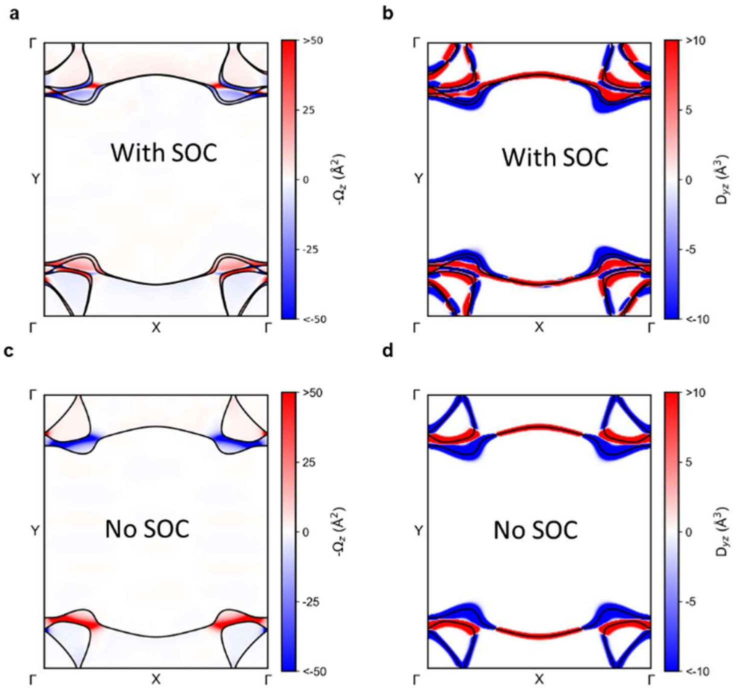

Fig. S23: Effect of SOC on Berry curvature and BCD distributions. Berry curvature distribution (a) with SOC and (c) without SOC. BCD distribution (b) with SOC and (d) without SOC.

In addition, we performed a supplementary calculation on the (111)-$LaAlO_3$/$SrTiO_3$ heterostructure. The SOC-induced band splitting near the Fermi level reaches approximately 4.2 meV, while slightly larger than that the (112) case (see Fig. S24).

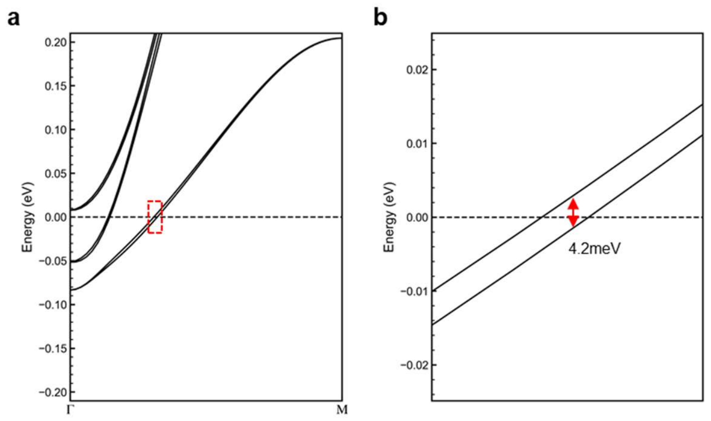

Fig. S24: SOC-induced band splitting near the Fermi level in the (111)-$LaAlO_3/SrTiO_3$ heterostructure. a, band structure along the Γ–X direction near the Fermi level. The red dashed box highlights the region of interest. **b,** Magnified view of the boxed region in (a), showing a spin-orbit-induced band splitting of approximately 4.2 meV near the Fermi energy, larger than that of (112)-$LaAlO_3/SrTiO_3$.

Supplementary Note 16: Estimation of Berry curvature dipole via experimental data and DFT

We further infer the magnitude of the Berry curvature dipole based on the value of $C_{in} = 57$ at low temperature (See Supplementary Note 10). To this end, we employ the formula $D_{yz} = \frac{V_{xy}^{2\omega}\sigma_{xx}^3 W}{(I_{xx}^{\omega})^2}\frac{2\hbar^2}{e^3\tau} = C_{in}\sigma_{xx}\frac{2\hbar^2}{e^3\tau}$, where τ is the relaxation time, $\hbar$ is the Dirac constant I_{xx}^{ω} is the sourced current, $V_{xy}^{2\omega}$ is the nonlinear Hall voltage, σ_{xx} is the (sheet) longitudinal conductivity, W is the Hall bar width[12]. We estimate the τ by calculating $\tau = \frac{\mu_{\mathrm{H}} m^*}{e}$, where m^* effective mass, μ_{H} is the Hall mobility. For convenience, we adopt m^* of (111)-$LaAlO_3/SrTiO_3$ reported[12], that is 3.49 m_e, then we have τ at the 1.55 K about 1.0 ps. Using the NLHE coefficient obtained, we estimated BCD to be ~440 nm at 1.55 K. The BCD magnitude derived from experimental results is about one order of magnitude larger than one of the (111)-$LaAlO_3/SrTiO_3$ interface at low temperature (< 30 K).

There is a gap between the BCD magnitude derived from experimental data and that from the DFT calculation. In fact, it is common that the nonlinear transport coefficient from DFT calculations are smaller than experimental result[12,17,18,21].

In our case, to perform DFT calculations for the (112) high-index interface, we constructed a superlattice structure containing 60 atoms, that are much larger than the typical unit cells used in common studies, which usually include only a few or a dozen atoms[21-23]. While the structure is

tractable for conventional DFT tasks such as structural relaxation and band analysis, the BCD calculation is considerably more challenging, as it requires extremely dense k-space sampling to achieve convergence due to the sensitivity to fine features near the Fermi level. To overcome this difficulty, we employed Wannier interpolation and carried out BCD results based on a 1000×1000 k-point mesh. We note that constructing well-localized Wannier functions for such a large supercell is itself a nontrivial task, requiring careful disentanglement and projection procedures.

Moreover, the 2D electron gas in the interface partially comes from the $3d$ orbitals in $SrTiO_3$, for which the correlation effect can be large. This correlation effect cannot be well captured by the DFT calculation. Modelling the band scheme in theory is further complicated by ill-defined oxygen stoichiometries and high dielectric permittivity[24]. Nevertheless, our DFT calculation still represents a state-of-the-art theoretical calculation of this newly developed system, offering valuable insight into its band structure, distribution of geometrical features in k-space and Fermi energy dependence of the BCD. We hope future work would bridge the gap between the DFT and experimental characterization.

Supplementary References

1 Son, J., Kim, K.-H., Ahn, Y., Lee, H.-W. & Lee, J. Strain engineering of the Berry curvature dipole and valley magnetization in monolayer MoS_2. *Phys. Rev. Lett.* **123**, 036806 (2019).

2 Delugas, P. *et al.* Spontaneous 2-dimensional carrier confinement at the *n*-type $SrTiO_3/LaAlO_3$ interface. *Phys. Rev. Lett.* **106**, 166807 (2011).

3 Gabel, J. *et al.* Interface band engineering in $LaAlO_3/SrTiO_3$ heterostructures. *Phys. Rev. B* **108**, 045125 (2023).

4 Annadi, A. *et al.* Anisotropic two-dimensional electron gas at the $LaAlO_3/SrTiO_3$ (110) interface. *Nat. Commun.* **4**, 1838 (2013).

5 Herranz, G., Sánchez, F., Dix, N., Scigaj, M. & Fontcuberta, J. High mobility conduction at (110) and (111) $LaAlO_3/SrTiO_3$ interfaces. *Sci. Rep.* **2**, 1 (2012)

6 Kang, K. F., Li, T. X., Sohn, E., Shan, J. & Mak, K. F. Nonlinear anomalous Hall effect in few-layer WTe_2 *Nat. Mater.* **18**, 324-328 (2019).

7 Kumar, D.*, et al.* Room-temperature nonlinear Hall effect and wireless radiofrequency rectification in Weyl semimetal $TaIrTe_4$. *Nat. Nanotechnol.* **16**, 421-425 (2021).

8 Lu, X. F. *et al.* Nonlinear transport and radio frequency rectification in BiTeBr at room temperature. *Nat. Commun.* **15**, 1 (2024).

9 He, P. *et al.* Quantum frequency doubling in the topological insulator Bi_2Se_3. *Nat. Commun.* **12**, 698 (2021).

10 Zhai, J. F. *et al.* Large nonlinear transverse conductivity and Berry curvature in $KTaO_3$ based two-dimensional electron gas. *Nano Lett.* **23**, 11892 (2023).

11 Du, Z. Z., Wang, C. M. X., Li, S., Lu, H. Z. & Xie, X. C. Disorder-induced nonlinear Hall effect with time-reversal symmetry. *Nat. Commun.* **10** 3047 (2019).

12 Lesne, E.*, et al.* Designing spin and orbital sources of Berry curvature at oxide interfaces. *Nat. Mater.* **22**, 576-

582 (2023).

13 Ohuchi, Y. *et al.* Electric-field control of anomalous and topological Hall effects in oxide bilayer thin films. *Nat. Commun.* **9**, 213 (2018).

14 Dugaev, V., Bruno, P., Taillefumier, M., Canals, B. & Lacroix, C. Anomalous Hall effect in a two-dimensional electron gas with spin-orbit interaction. *Phys. Rev. B* **71**, 224423 (2005).

15 Duan, J., *et al.* Giant second-order nonlinear Hall effect in twisted bilayer graphene. *Phys. Rev. Lett.* **129**, 186801 (2022).

16 Huang, M. Z. *et al.* Intrinsic nonlinear Hall effect and gate-switchable Berry curvature sliding in twisted bilayer graphene. *Phys. Rev. Lett.* **131** (2023).

17 Ma, Q., *et al.* Observation of the nonlinear Hall effect under time-reversal-symmetric conditions. *Nature* **565**, 337-342 (2019).

18 Mercaldo, M. T., Noce, C., Caviglia, A. D., Cuoco, M. & Ortix, C. Orbital design of Berry curvature: pinch points and giant dipoles induced by crystal fields. *npj Quantum Mater.* **8**, 12 (2023).

19 Johansson, A., Göbel, B., Henk, J., Bibes, M. & Mertig, I. Spin and orbital Edelstein effects in a two-dimensional electron gas: Theory and application to $SrTiO_3$ interfaces. *Phys. Rev. Res.* **3**, 013275 (2021).

20 El Hamdi, A. *et al.* Observation of the orbital inverse Rashba–Edelstein effect. *Nat. Phys.* **19**, 1855-1860 (2023).

21 Wang, H. & Qian, X. Ferroelectric nonlinear anomalous Hall effect in few-layer WTe_2. *npj Quantum Mater.* **5**, 119 (2019).

22 You, J.-S., Fang, S., Xu, S.-Y., Kaxiras, E. & Low, T. Berry curvature dipole current in the transition metal dichalcogenides family. *Phys. Rev. B* **98**, 121109 (2018).

23 Wang, E., Zeng, H., Duan, W. & Huang, H. Spontaneous inversion symmetry breaking and emergence of Berry curvature and orbital magnetization in topological $ZrTe_5$ films. *Phys. Rev. Lett.* **132**, 266802 (2024).

24 Gabel, J. *et al.* Toward functionalized ultrathin oxide films: the impact of surface apical oxygen. *Adv. Electron. Mater.* **8**, 2101006 (2022).
